# Preparation and Characterization of Novel Cationic Chitosan Derivatives Bearing Quaternary Ammonium and Phosphonium Salts and Assessment of Their Antifungal Properties

**DOI:** 10.3390/molecules22091438

**Published:** 2017-08-31

**Authors:** Wenqiang Tan, Qing Li, Fang Dong, Qiuhong Chen, Zhanyong Guo

**Affiliations:** 1 Key Laboratory of Coastal Biology and Bioresource Utilization, Yantai Institute of Coastal Zone Research, Chinese Academy of Sciences, Yantai 264003, China; wqtan@yic.ac.cn (W.T.); qli@yic.ac.cn (Q.L.); fdong@yic.ac.cn (F.D.); qhchen@yic.ac.cn (Q.C.); 2 University of Chinese Academy of Sciences, Beijing 100049, China

**Keywords:** cationic chitosan derivatives, quaternary ammonium salts, quaternary phosphonium salts, antifungal activity, electron-withdrawing ability

## Abstract

Chitosan is an abundant and renewable polysaccharide, its derivatives exhibit attractive bioactivities and the wide applications in various biomedical fields. In this paper, two novel cationic chitosan derivatives modified with quaternary phosphonium salts were successfully synthesized via trimethylation, chloride acetylation, and quaternization with tricyclohexylphosphine and triphenylphosphine. The structures and properties of synthesized products in the reactions were characterized by FTIR spectroscopy, ^1^H-NMR, ^31^P-NMR, elemental and thermogravimetric analysis. The antifungal activities of chitosan derivatives against four kinds of phytopathogens, including *Phomopsis asparagi*, *Watermelon fusarium*, *Colletotrichum lagenarium*, and *Fusarium oxysporum* were tested using the radial growth assay in vitro. The results revealed that the synthesized cationic chitosan derivatives showed significantly improved antifungal efficiency compared to chitosan. It was reasonably suggested that quaternary phosphonium groups enabled the obviously stronger antifungal activity of the synthesized chitosans. Especially, the triphenylphosphonium-functionalized chitosan derivative inhibited the growth of *Phomopsis asparagi* most effectively, with inhibitory indices of about 80% at 0.5 mg/mL. Moreover, the data demonstrated that the substituted groups with stronger electron-withdrawing ability relatively possessed greater antifungal activity. The results suggest the possibility that cationic chitosan derivatives bearing quaternary phosphonium salts could be effectively employed as novel antifungal biomaterials for application in the field of agriculture.

## 1. Introduction

Chitosan, a naturally occurring linear biopolymer composed of d-glucosamine and *N*-acetyl-d-glucosamine residues, is derived from the complete or partial deacetylation of chitin [1,2]. Chitosan has a unique set of interesting characteristics such as biocompatibility, biodegradability, non-toxicity, and antimicrobial activity [3,4,5], and this has led to its wide applications in pharmaceuticals, cosmetics, agriculture, food science, and textiles [3,6,7]. However, its poor solubility in both organic and aqueous solvents limits considerably the further applications of chitosan in various advanced biomedical fields [6,8,9]. Chemical modification or derivatizations of chitosan by introducing small functional groups into the chitosan backbone to increase its solubility at neutral and alkaline pH values as well as enhance its original bioactivities have attracted special attention [8,10,11]. Moreover, the active primary amine and hydroxyl groups on the chitosan backbone provide excellent reactive sites for chemical modifications.

Of all the chitosan derivatives, cationic chitosans have many unique important physicochemical features such as water solubility and biological properties, especially antimicrobial activity [1,12]. As a most typical cationic chitosan, quaternary ammonium-functionalized chitosan derivatives have attracted increasing interest from both an academic and industrial point of view [12,13,14]. Among them, *N*,*N*,*N*-trimethylchitosan with good water solubility can be synthesized rapidly and effectively by peralkylation of free amine groups in the chitosan molecule with excess iodomethane [12,15,16], and have special appeal as substrates for the introduction of the other functional groups into the chitosan backbone. Recently, the compounds bearing quaternary phosphonium salts were reported to exhibit a higher antimicrobial activity compared with those with quaternary ammonium salts [17,18,19]. Quaternary phosphonium salts are considered as a new generation of efficient and broad-spectrum antiseptics [20,21]. The modification of chitosan by quaternary phosphonium salts can help enhance the biological activity and application value of chitosan. Guo et al. described that *N*-triphenylphosphonium chitosans with different degrees of substitution had favorable antibacterial activity and could be used as potential polymeric antibacterial agents [22]. Qiao et al. developed low toxicity *N*-triphenylphosphonium chitosans with two degrees of substitution which could be used as effective gene delivery vectors [23]. However, to the best of our knowledge these works have only focused on one kind of quaternary phosphonium salts—triphenylphosphonium salts. Besides, the previous studies mainly reported the antibacterial action of quaternary phosphonium-functionalized chitosan derivatives. Our group has reported the synthesis and antifungal property of low molecular weight chitosan derivatives bearing quaternary phosphonium groups, and chloroacetyl chitosan prepared by the reaction of low molecular weight water-soluble chitosan and chloroacetyl chloride in aqueous solutions was used as reaction intermediate [24], but this method is not a good fit for high molecular weight chitosan because of the poor water solubility of this substrate.

The aim of our project was to prepare the chitosan derivatives bearing quaternary phosphonium groups using high molecular weight chitosan as original raw material and investigate their antifungal activity. In this paper, we hereby report the preparation and antifungal property of two chitosan derivatives modified with quaternary phosphonium salts using high molecular weight chitosan as original raw material. The chemical structures of the derivatives were characterized by FTIR, ^1^H-NMR, and ^31^P-NMR. The quantitative data on degree of substitution, thermal stability, and water solubility of the synthesized chitosan derivatives were calculated. Four plant-threatening fungi, *Phomopsis asparagi* (*P. asparagi*), *Watermelon fusarium* (*W. fusarium*), *Colletotrichum lagenarium* (*C. lagenarium*), and *Fusarium oxysporum* (*F. oxysporum*), were selected to evaluate the antifungal properties of the new derivatives by hypha measurement in vitro.

## 2. Results and Discussion

### 2.1. Chemical Synthesis and Characterization

**Scheme 1:**
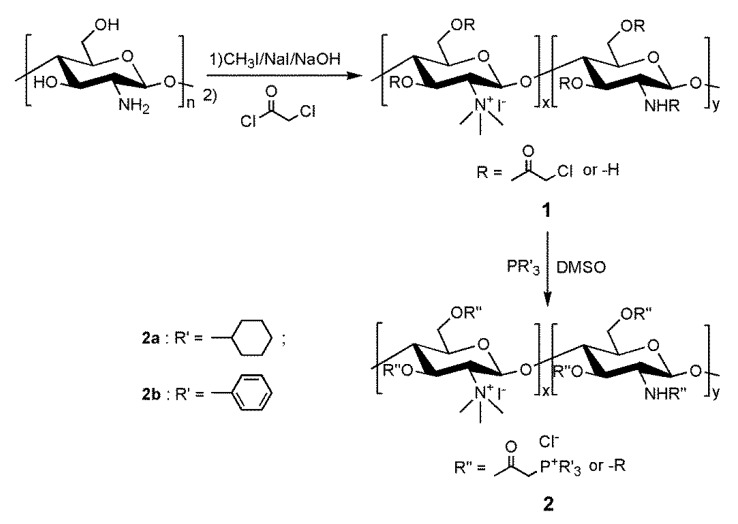
Synthetic route to the novel chitosan derivatives.

**Figure 1:**
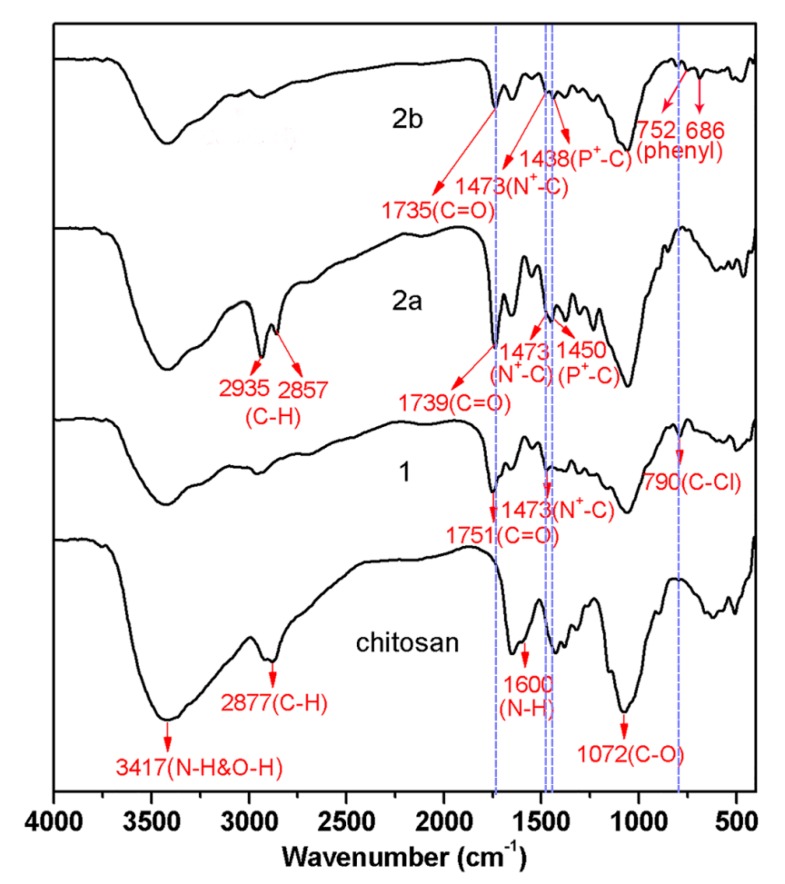
FTIR spectra of chitosan and chitosan derivatives.

**Figure 2:**
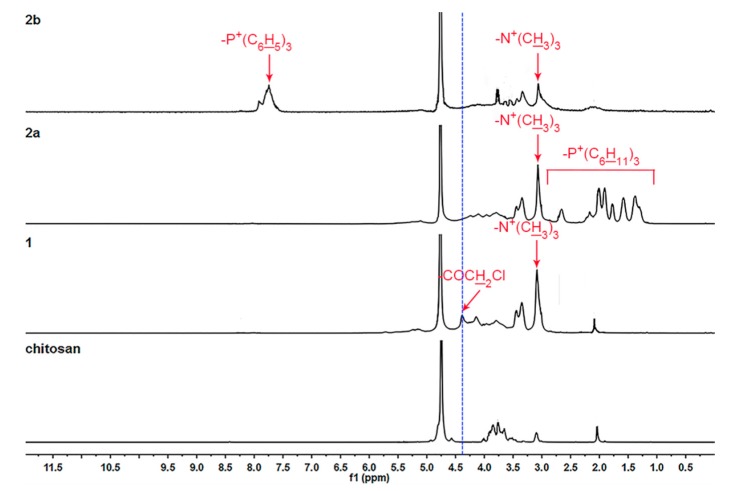
^1^H-NMR spectra of chitosan and chitosan derivatives.

**Figure 3:**
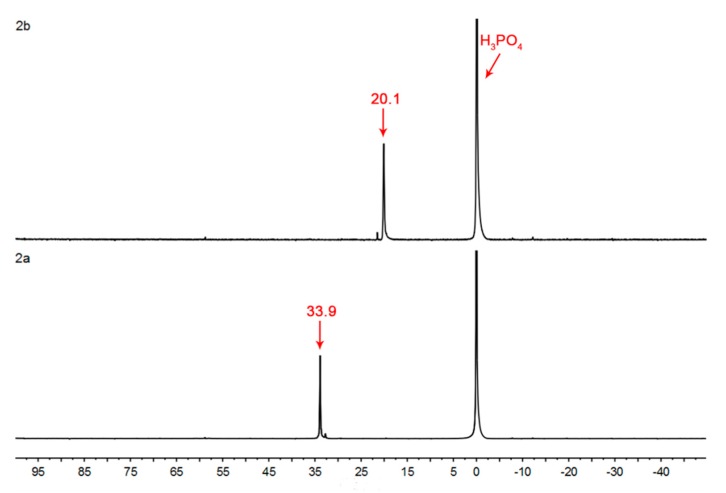
^31^P-NMR spectra of chitosan derivatives.

The synthetic strategy for the preparation of high molecular weight chitosan derivatives bearing quaternary phosphonium salts is shown in Scheme 1. Considering the poor solubility of pristine chitosan in water and organic solvents, trimethylation of amino groups as one of the most simple and efficient methods to avoid inefficient heterogeneous reaction conditions in the next chemical step [12,15]. Without any purification, *N*,*N*,*N*-trimethylchitosan can directly react with chloroacetyl chloride to obtain the reaction intermediate chloracetyl chitosan bearing quaternary ammonium salt **1**. Then, the targeted chitosan derivatives **2a** and **2b** were synthesized by a one step chemical approach, by reacting chitosan derivative **1** with tricyclohexylphosphine and triphenylphosphine, respectively. In order to verify the successful synthesis of each compound, the FTIR (Figure 1) and ^1^H-NMR (Figure 2) spectra of chitosan and chitosan derivatives and ^31^P-NMR (Figure 3) spectra of the chitosan derivatives were recorded.

A comparison of the FTIR spectra for chitosan and the chitosan derivatives is given in Figure 1. The FTIR spectrum of chitosan displays a characteristic peak at 3417 cm^−1^, which is attributed to the –NH_2_ and –OH stretching vibration [1,8,9], at 2877 cm^−1^, which is attributed to the –CH_2_– stretching vibration [25], at 1600 cm^−1^, which is assigned to the –NH_2_ bending vibration [10,12,26], and at 1072 cm^−1^, which is assigned to the –C–O–C– stretching vibration of the glucosamine ring [8]. The formation of compound **1** is confirmed by the peaks at 1751 and 790 cm^−1^ in the FTIR spectrum which are due to the vibration absorption of –C=O and –C–Cl in the chloroacetyl group [27,28,29]. In addition, the peak at 1473 cm^−1^ is assigned to –N^+^(CH_3_)_3_ stretching vibrations [11,15]. After the quaternization reactions of compound **1** with tricyclohexylphosphine and triphenylphosphine, the peak of –C–Cl at 790 cm^−1^ is nearly eliminated and characteristic bands at 1438–1450 cm^−1^ assigned to the –P^+^R_3_ groups (R = cyclohexyl or phenyl) appear [30]. Besides, it is worth noting that the peak of –C=O stretching vibration of carboxylic esters at 1751 cm^−1^ has shifted to 1735 or 1739 cm^−1^ which may be attributed to the electron-withdrawing inductive effect of the quaternary phosphonium groups [22]. In addition, new peaks appear at 2935 and 2857 cm^−1^ (–CH– stretching vibration of cyclohexyl in compound **2a**), and 752 and 686 cm^−1^ (–CH– bending vibration of phenyl in compound **2b**) in the spectra of chitosan derivatives **2**, indicating that tricyclohexylphosphine and triphenylphosphine have been grafted into the chitosan backbone.

The ^1^H-NMR spectra were further applied to confirm the chemical structures of chitosan and chitosan derivatives. As shown in Figure 2, the signals at 3.0–5.0 ppm are assigned to the protons of glucosamine unit of chitosan and the peak at 2.0 ppm is assigned to the methyl protons of the *N*-acetyl group [7,11]. Compared with the peaks of chitosan, the ^1^H-NMR spectrum of compound **1** shows a prominent –N^+^(CH_3_)_3_ peak at 3.1 ppm [31,32] along with the resonance peak at 4.4 ppm corresponding to the methylene protons of –COCH_2_Cl group [27,29]. Compared to compound **1**, it can be clearly seen from the ^1^H-NMR spectra of chitosan derivatives **2a** and **2b** in Figure 2 that the characteristic resonance at 4.4 ppm for –COCH_2_Cl weakens greatly while new signals appear at 1.2-2.7 ppm in compound **2a**, which are assigned to the protons of methyl and methylene moieties, and the resonances at 7.5–8.0 ppm are responded to the phenyl protons in compound **2b** [18,32].

The ^31^P-NMR spectra of chitosan derivatives bearing quaternary ammonium and phosphonium salts are shown in Figure 3. The characteristic peaks at 33.9 and 20.1 ppm are attributed to the phosphorus atoms of the various quaternary phosphonium groups in chitosan derivatives **2a** and **2b**, respectively.

### 2.2. Thermal Stability

**Figure 4:**
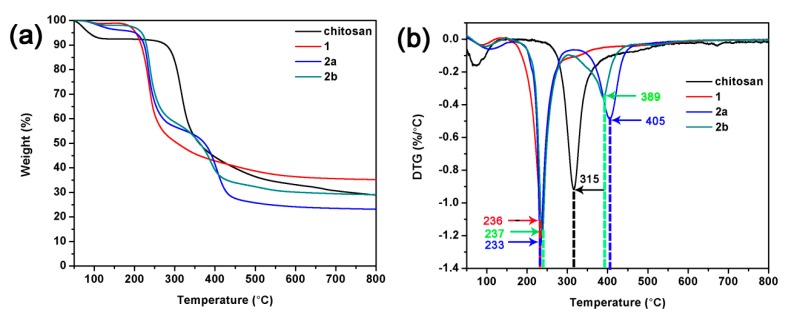
TGA (**a**) and DTG (**b**) curves of chitosan and chitosan derivatives.

The thermogravimetric analyses (TGA) and derivative thermogravimetric (DTG) analyses curves of chitosan and chitosan derivatives are shown in Figure 4. They show that chitosan and chitosan derivative **1** have two stages of weight loss, while chitosan derivatives **2a** and **2b** undergo a three-step weight loss under a nitrogen atmosphere. The initial weight loss (below 150 °C) observed in all samples is due to the loss of absorbed and bound water. The major weight loss of chitosan occurs from 280 °C to 350 °C and 68% of mass is decomposed up to 800 °C. In contrast to chitosan, chitosan derivatives undergo a major weight loss from about 190 °C to 300 °C and 65–71% of their masses are decomposed up to 800 °C. In the second stage, the temperatures at the maximum degradation rate values of chitosan and chitosan derivatives **1**, **2a** and **2b** are 315, 236, 233 and 237 °C, respectively, which indicates that chemical modification has a significant influence on the thermal stability of chitosan which may be due to the introduction of functional groups, which can break the intramolecular and intermolecular hydrogen bonding interaction and destroy the crystalline structure of the chitosan molecule [31,33,34]. Besides, in the third stage, the temperatures at the maximum degradation rate values registered at 405 °C and 389 °C for chitosan derivatives **2a** and **2b**, respectively.

### 2.3. Water Solubility

**Figure 5:**
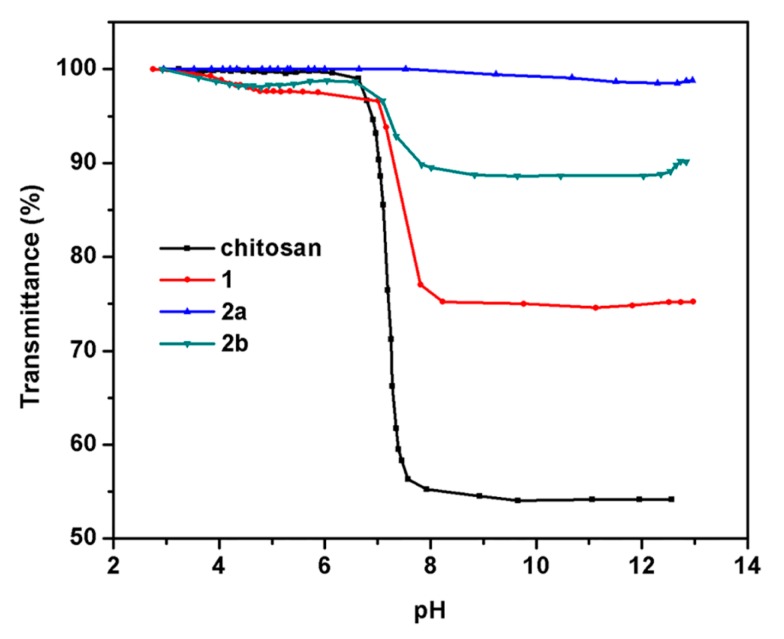
pH dependence of water solubility of chitosan and chitosan derivatives.

Figure 5 illustrates the water solubility of chitosan and chitosan derivatives at different pH values. It is well established that chitosan has poor solubility in water due to its strong intramolecular and intermolecular hydrogen bonds [10]. It is highly soluble in acidic solutions (pH < 6.5) due to the protonation of the primary amine [12,35]. After trimethylation of chitosan, chitosan derivative **1** showed higher water solubility because of the high hydrophilicity of its quaternary ammonium groups [16,31]. Meanwhile, the introduction of quaternary phosphonium moieties into chitosan derivative **1** further improves the water solubility, especially at alkaline pH (pH > 8.0). Table 1 also shows that the chitosan derivatives produced are soluble in 1% HOAc aqueous solution, water, DMSO, and DMF. The current finding suggest that the quaternization reaction is an effective mean for improving water solubility of chitosan, which is favorable to the application of chitosan.

### 2.4. Antifungal Activity

**Figure 6:**
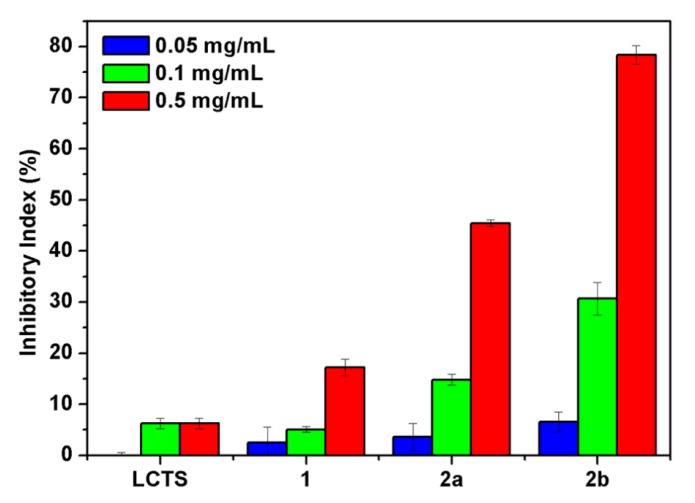
The antifungal activity of chitosan and chitosan derivatives against *P. asparagi*.

**Figure 7:**
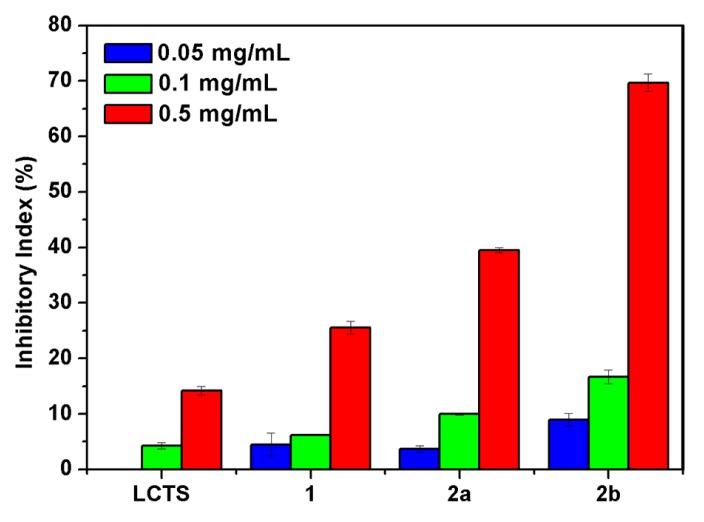
The antifungal activity of chitosan and chitosan derivatives against *W. fusarium*.

**Figure 8:**
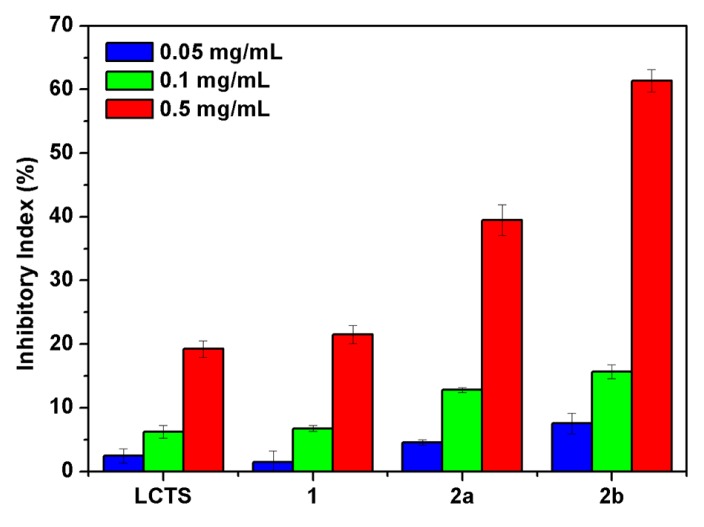
The antifungal activity of chitosan and chitosan derivatives against *C. lagenarium*.

**Figure 9:**
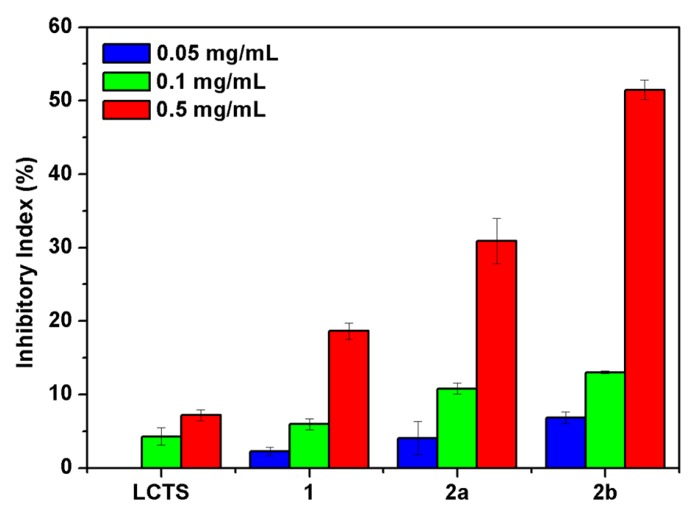
The antifungal activity of chitosan and chitosan derivatives against *F. oxysporum*.

Four destructive phytopathogenic fungi, *P. asparagi*, *W. fusarium*, *C. lagenarium*, and *F. oxysporum*, were applied to test the potential antifungal activity of the prepared compounds in vitro by measuring mycelial inhibition of radial growth. The capabilities of the chitosan derivatives to inhibit the growth of the tested four common plant-threatening fungi are shown in Figure 6, Figure 7, Figure 8 and Figure 9, respectively. The growth-inhibiting effect was quantitatively determined by the ratio of the diameter of the growth zone in the medium with the chitosan derivatives to those with the deionized water instead of the chitosan derivatives (control).

*P. asparagi* is a kind of destructive pathogenic fungus that can cause severe stem blight of asparagus. As it can be seen from the Figure 6, the inhibitory indices of all of the compounds enhanced with the increase of concentration. All of the synthesized products show better ability of inhibiting the growth of *P. asparagi* than chitosan, and the highest antifungal activity is observed at 0.5 mg/mL. Compared with chitosan and compound **1**, with inhibitory indices of 6.25% and 17.21%, the inhibitory indices of compounds **2a** and **2b** are 45.45% and 78.35%, respectively, which suggests that quaternary phosphonium groups should be the antifungal function groups. Furthermore, compound **2b** is more active than compound **2a** against *P. asparagi*. It is evident that the antifungal activity against *P. asparagi* decreases in the order: **2b** > **2a** > **1** > chitosan. Notably, compound **2b** is still active against *P. asparagi* with inhibitory values of over 30% even when the dosage is lowered to 0.1 mg/mL.

*W. fusarium* can cause severe fusarium wilt and foot rot of some fruit plants, such as watermelon. As shown in Figure 7, all the samples are active against *W. fusarium* to some extent, and the antifungal activity of chitosan derivatives is concentration-dependent. The inhibitory indices of chitosan, compounds **1**, **2a** and **2b** against *W. fusarium* are 14.20%, 25.50%, 39.49% and 69.66% at 0.5 mg/mL, respectively. The antifungal strong-to-weak sequence of the compounds against *W. fusarium* is similar to that against *P. asparagi*. The rationale behind the increased antifungal activity of compounds **2a** and **2b** versus compound **1** can be the result of the introduction of quaternary phosphonium groups which can contribute a lot to the antifungal action and consequently increased the antifungal activity of them.

*C. lagenarium*, carried mainly by ungerminated seeds, can lead to serious consequences for cucumber leaves that turn brown and wither. *F. oxysporum* can cause severe root rot and even death of the whole plant by infecting plant roots. Figure 8 and Figure 9 show the antifungal activity of chitosan and all the derivatives against *C. lagenarium* and *F. oxysporum*. The inhibitory indices of all the samples increase with increasing concentration. The chitosan derivatives **2** exhibit evidently better antifungal activity than chitosan and compound **1**. The results reconfirm the significant contribution of quaternary phosphonium groups to the antifungal activity of the synthesized chitosan derivatives. Moreover, compared with compound **2a** with inhibitory indices of 39.45% and 30.91%, the inhibitory indices of compound **2b** are 61.36% and 51.48% at 0.5 mg/mL against *C. lagenarium* and *F. oxysporum*.

Based on the results mentioned above, the synthesized quaternary ammonium and phosphonium functionalized chitosan derivatives showed evidently enhanced antifungal properties against the four tested plant pathogenic strains at the tested concentrations compared with chitosan. The effective antifungal properties should be ascribed to the quaternary phosphonium groups. These quaternary phosphonium-functionalized chitosan derivatives could target the plant pathogens via the electrostatic interactions of amphiphile molecules on the outer cellular membranes [11,32]. The positively charged moieties of the cationic chitosan derivatives could interact with the negatively charged components on the fungal cell walls or cytomembranes [32,36,37]. This adherence of polycations to the outer membranes of the fungi could disrupt the microbial cell surface to hinder the transport of essential nutrients into the cell and also cause severe leakage of the cell constituents [11,38,39]. Generally, taking into consideration the relationships between inhibitory indices and structural characteristic of chitosan derivatives, electron-withdrawing substituent groups in quaternary phosphonium groups might be the major effective factor. The inhibition prowess of quaternary phosphonium functionalized chitosan derivatives against plant pathogens generally increases with the increasing electron-withdrawing ability of the substituent groups. In particular, strong electron-withdrawing groups could strengthen the positive charge density of quaternary phosphonium groups and could also more easily result in cell death [12].

## 3. Experimental Section

### 3.1. Materials

Chitosan with degree of deacetylation of 0.83 (C: 43.42%, N: 7.98%, H: 6.30%, C/N: 5.44) and molecular weight of 200 kDa was purchased from Qingdao Baicheng Biochemical Corp. (Qingdao, China). Iodomethane, chloroacetyl chloride, tricyclohexylphosphine (TCP), and triphenylphosphine (TPP) were purchased from the Sigma-Aldrich Chemical Corp. (Shanghai, China). The other reagents were all analytical grade and used as received. Four plant-threatening fungi, *P. asparagi*, *W. fusarium*, *C. lagenarium*, and *F. oxysporum*, were obtained from the Qingdao Academy of Agricultural Sciences.

### 3.2. Structural Characterization of Chitosan Derivatives

Fourier transform infrared (FTIR) spectra were recorded on a Jasco-4100 Fourier Transform Infrared Spectrometer (Jasco Co., Ltd., Shanghai, China) at 25 °C in the transmittance mode. The tested samples were grinded and mixed with KBr in the weight ratio 1/100 for observations. All spectra were scanned against a blank KBr pellet back-ground in the range of 4000–400 cm^−1^ at a resolution of 4.0 cm^−1^.

^1^H nuclear magnetic resonance (^1^H-NMR) spectra were recorded on an AVIII-500 spectrometer (Bruker, Zurich, Switzerland, provided by Bruker Tech. and Serv. Co., Ltd., Beijing, China) at 25 °C using D_2_O as solvent. Chemical shifts (δ ppm) were referenced to tetramethylsilane (TMS).

^31^P nuclear magnetic resonance (^31^P-NMR) spectra were measured with a Bruker AVANCE III spectrometer (provided by Bruker Tech. and Serv. Co., Ltd., Beijing, China) at room temperature operating at 600 MHz. Chemical shifts were recorded in parts per million relative to 85% of H_3_PO_4_ (0.0 ppm) in solution.

The thermogravimetric analyses (TGA) of samples were performed using a Mettler 5 MP thermal analyzer (Mettler-Toledo, Zurich, Switzerland). The samples were heated from 50 °C to 800 °C at a heating rate of 10 °C/min under a nitrogen flow.

The elemental analyses (C, H, and N) were carried out using a Vario EL III (Elementar, Langenselbold, Germany). The degrees of substitution (DS) of chitosan derivatives were calculated on the basis of the percentages of carbon and nitrogen according to the following equations:

(1) $$DS_{1}=\frac{n_{1}\times M_{C}-M_{N}\times W_{1}}{n_{2}\times M_{C}}$$

(2) $$DS_{2}=\frac{M_{N}\times W_{2}+n_{2}\times M_{C}\times DS_{1}-n_{1}\times M_{C}}{n_{3}\times M_{C}}$$

(3) $$DS_{3}=\frac{M_{N}\times W_{3}+n_{2}\times M_{C}\times DS_{1}-n_{1}\times M_{C}-n_{3}\times M_{C}\times DS_{2}}{n_{4}\times M_{C}}$$

(4) $$DS_{4}=\frac{M_{N}\times W_{4}+n_{2}\times M_{C}\times DS_{1}-n_{1}\times M_{C}-n_{3}\times M_{C}\times DS_{2}-n_{4}\times M_{C}\times DS_{3}}{n_{5}\times M_{C}}$$

where *DS*_1_, *DS*_2_, *DS*_3_, and *DS*_4_ represent the deacetylation degree of chitosan, the degrees of substitution of *N*,*N*,*N*-trimethyl in chitosan derivatives, chloroacetyl in chitosan derivative **1**, and quaternary phosphonium groups in chitosan derivatives **2**; *M*_C_ and *M*_N_ are the molar mass of carbon and nitrogen, *M*_C_ = 12, *M*_N_ = 14; *n*_1_, *n*_2_, *n*_3_, *n*_4_, and *n*_5_ are the number of carbon of chitin, acetamido group, trimethyl, chloroacetyl group, and tricyclohexylphosphine or triphenylphosphine, *n*_1_ = 8, *n*_2_ = 2, *n*_3_ = 3, *n*_4_ = 2, *n*_5_ = 18; *W*_1_, *W*_2_, *W*_3_, and *W*_4_ represent the mass ratio (C/N ratio) between carbon and nitrogen in chitosan, *N*,*N*,*N*-trimethyl chitosan, chitosan derivative **1**, and chitosan derivatives **2**.

### 3.3. Synthesis of Chitosan Derivatives

#### 3.3.1. Synthesis of Chloracetyl Chitosan Derivative with Quaternary Ammonium Salt **1**

Chitosan (0.322 g, 2 mmol of glucosamine) was dispersed in *N*-methyl-2-pyrrolidone (NMP, 15 mL) and stirred at room temperature for 1 h. Then, NaI (0.90 g, 6 mmol), 15% NaOH aqueous solution (3 mL, 11 mmol), and CH_3_I (3 mL, 48 mmol) were subsequently added. The mixture was allowed to warm up to 60°C and stirred under reflux for an additional 1 h. The reaction solution was poured into 150 mL of absolute ethanol to afford some flavescent precipitate (elemental analysis: C: 31.43%, N: 4.52%, H: 5.49%, C/N: 6.95, DS_trimethyl_: 0.59). The precipitate collected by filtration was then dissolved in 30 mL of NMP and stirred at room temperature for 1 h before chloracetyl chloride (0.3 mL, 4 mmol) was added dropwise. After stirring continuously at room temperature for 12 h, the solution was poured into 150 mL of absolute ethanol to produce a yellowish precipitate. The precipitate was collected by filtration and then washed with ethanol for three times carefully. The resultant product was obtained by freeze-drying overnight in vaccum. Chitosan derivative **1**: Yield: 71.1%; Elemental analysis: C: 36.32%, N: 4.35%, H: 5.81%, C/N: 8.35, DS_chloroacetyl_: 0.82.

#### 3.3.2. Synthesis of Chitosan Derivatives Bearing Quaternary Ammonium and Phosphonium Salts **2a** and **2b**

Chloracetyl chitosan derivative with quaternary ammonium salt **1** (0.421 g) was dissolved in anhydrous dimethyl sulfoxide (DMSO, 15 mL) in a 50 mL flask, then organic phosphines (3 mmol) were added to the flask and dissolved, respectively. The solution was heated to 60 °C and stirred for 24 h under nitrogen. Then, the reaction solution was poured into 150 mL of acetone and collected by filtration, washed carefully with acetone. The unreacted organic phosphines were extracted in a Soxhlet apparatus with acetone for 24 h. The final products were obtained by drying in vacuum freeze dryer. Chitosan derivative **2a**: Yield: 73.2%; Elemental analysis: C: 47.60%, N: 3.62%, H: 7.88%, C/N: 13.15, DS_tricyclohexylphosphonium_: 0.31. Chitosan derivative **2b**: Yield: 70.8%; Elemental analysis: C: 46.63%, N: 3.87%, H: 6.41%, C/N: 12.05, DS_triphenylphosphonium_: 0.24.

### 3.4. Estimation of Water Solubility

The water solubility of chitosan and chitosan derivatives at various pH values was determined by a turbidity measurement [40]. Briefly, 0.1 g of chitosan was dissolved in 100 mL of 1% HOAc aqueous solution and subsequently the transmittance of the solution at different pH values was recorded with the stepwise addition of 1 M NaOH on a TU-1810 UV spectrometer (General Instrument Co., Ltd., Beijing, China) at 600 nm.

### 3.5. Antifungal Assay

In consideration of the poor water solubility of pristine chitosan, the water-soluble low molecular weight chitosan (LCTS) was used for antifungal activity tests. All tested compounds (LCTS and chitosan derivatives **1**, **2a** and **2b**) were firstly dissolved in distilled water at a concentration of 5 mg/mL as stock solutions. Then, each sample solution was added to sterile PDA medium to give the final concentrations of 0.05, 0.1 and 0.5 mg/mL. The final solutions were poured into sterilized Petri dishes (9 cm). After solidification, a mycelia disk (diameter: 5 mm) of active fungi was transferred to the center of the PDA Petri dishes and inoculated at 27 °C. When the mycelia of fungi reached the edges of the control plate (without the presence of samples), the growth inhibition was calculated by the formula:

(5) $$\mathrm{Inhibitory}\;\mathrm{index}\;(\%)=(1-D_{a}/D_{b})\times 100$$

where *D_a_* is the diameter of the growth zone in the test plates and *D_b_* is the diameter of the growth zone in the control plate.

### 3.6. Statistical Analysis

All the experiments were performed in triplicate and the data were expressed as mean ± the standard deviation (SD, *n* = 3). Significant difference analysis was determined using Scheffe’s multiple range test. A level of *p* < 0.05 was considered statistically significant.

## 4. Conclusions

In this paper, cationic chitosan derivatives containing quaternary ammonium and phosphonium groups were successfully synthesized and their in vitro antifungal applications against four kinds of plant pathogens were reported. The quaternary ammonium- and phosphonium-functionalized chitosan derivatives were found to have significantly enhanced antifungal efficiency against plant pathogens when compared to chitosan. The results indicated that quaternary phosphonium groups should be high-efficiency antifungal groups. Moreover, the triphenylphosphonium-functionalized chitosan derivative showed the best antifungal activity and the electron-withdrawing ability of the different substituted groups on the quaternary phosphonium salts was believed to be the main reason for the marked improvement in antifungal behavior. They have the potential of becoming promising candidates for the control of fungal plant diseases and can also serve as a new lead structures for further design of antifungal materials.

## Figures and Tables

**Table 1:** Summary of the solubility properties of chitosan derivatives.

Compound	Solubility ^a^				
	H_2_O	1% HOAc aq.	DMSO	DMF	EtOH
Chitosan	−	+	−	−	−
**1**	+	+	+	+	−
**2a**	+	+	+	+	−
**2b**	+	+	+	+	−

^a^ (+) soluble, (−) insoluble.

## Abbreviations

The following abbreviation are used in this manuscript:

FTIR	Fourier transform infrared spectra
^1^H-NMR	^1^H nuclear magnetic resonance spectra
^31^P-NMR	^31^P nuclear magnetic resonance spectra
TGA	Thermogravimetric analyses
DTG	Derivative thermogravimetric
NMP	*N*-methyl-2-pyrrolidone
DMSO	Dimethyl sulfoxide
PDA	Potato dextrose agar
